# Management of Fruit Industrial By-Products—A Case Study on Circular Economy Approach

**DOI:** 10.3390/molecules25020320

**Published:** 2020-01-13

**Authors:** Débora A. Campos, Ricardo Gómez-García, Ana A. Vilas-Boas, Ana Raquel Madureira, Maria Manuela Pintado

**Affiliations:** CBQF—Centro de Biotecnologia e Química Fina—Laboratório Associado, Escola Superior de Biotecnologia, Universidade Católica Portuguesa, Rua Diogo Botelho 1327, 4169-005 Porto, Portugal; deborancampos@gmail.com (D.A.C.); ricky311091@gmail.com (R.G.-G.); aavilasboas.95@gmail.com (A.A.V.-B.); rmadureira@porto.ucp.pt (A.R.M.)

**Keywords:** antioxidant activity, circular economy, fruit by-products, value add molecules, industrial application, biorefinery concept, integral valorisation

## Abstract

The management of industrial fruit by-products is important not only to decrease the volume of food waste accumulated in the landfills but also to develop strategies through reuse with the purpose to valorise and add economic value. The disposal of food waste leads to different global issues in different sectors, such as social, environmental and economical. These by-products represent a rich source of valuable compounds (polyphenols) with high antioxidant activity, which can be extracted through biotechnological methodologies for future industrial applications. In this context, the management of fruit by-products is challenged to move from a linear economy to a circular economy. Therefore, the purpose of this review is to provide a critical view of an integrated valorisation of fruit by-products to overcome a global issue, via the production of antioxidant extracts with high economic value. A case study of pineapple processing industrialization in a circular economy is explored and discussed.

## 1. Introduction

Food waste is a social and economic issue due to excessive use of natural resources, which are limited and finite, leading to losses of millions of euros. Every day, the whole food system loses or wastes 30% of all produced foods, and all stakeholders have a responsibility to prevent these food losses. An overview should be performed of all systems to decrease the food losses in all responsible sectors, from producers and processors to intermediates until reaching the consumers [1].

For 2018 alone, the ScienceDirect database holds 3342 files of research regarding the circular economy. Regarding circular economy and fruit by-products, 437 publications were found, of which 73 were published in 2018 (25 reviews, 33 research papers, 7 book chapters, etc.). The most used keywords in this field were biorefinery, bio-economy and sustainable development. The journal that published the most regarding the present subject was the Journal of Cleaner Production. The circular economy has received considerable attention in the scientific field, as pioneers in the development of novel strategies towards the application of the circular economy in waste and resource management policy debate over the last few years.

In 2012, Europe produced 88 million tons of organic waste, representing 173 kg of organic waste per capita, and from the amount produced in industrial processing, 33 kg per capita of organic waste did not undergo any kind of recovery [2]. All this waste is generally released in landfills or burned for energy production leading to the loss of the remaining economic and biological value. Thus, strategies and projects are required to encourage upstream waste recovery, leading to the production of downstream value-added ingredients, always based on a sustainable economy, i.e., a circular economy. However, for an integrated recovery of success and considering the intricacies of food safety, several previous steps must be considered to ensure the future application of the resulting ingredients.

Fruits and vegetables are amongst the most widely consumed foods. The major contributors are China, USA and Philippines, who generate 32, 15 and 1.8 million tons of waste, respectively [3]. The different fruit wastes come from the production of different products, such as juices, juice concentrates, jams, canned fruit, dehydrated fruit and fresh cut fruit, beyond others, having a greatly negative impact in this field [4].

Once food security is assured all this waste can be valorised in an integrated way under the application of downstream processes by the industries, transforming the waste into secondary raw materials. Many molecules of interest can be extracted from the secondary raw materials of fruit by-products, and among these the most important economically (higher market value) include the enzymes and vitamins. However, after such extraction, other bioactive compounds can be recovered, such as phenolics, alkaloids, flavonoids, carotenoids, glycosides, tannins, saponins and terpenoids [5].

On the other hand, extracts can be used as functional ingredients due to their biological activities, such as antimicrobial, prebiotics and antioxidants, or be applied to produce functional food products [4]. In addition, the ingredients of cellulosic origin can be applied directly in non-food industries, such as paper and biodiesel industries, as well as sustainable packaging, which can help to reduce the environmental impact of secondary raw materials in the environment and increase market acceptance, when compared to those currently available in the market.

Thus, this review intends to give an overview of the circular economy, via the integrated valorisation of fruit by-products from food processing industries, which creates a potential opportunity for the extraction of value-added compounds, through application of sustainable and green methodologies.

## 2. Circular Economy—Essential to the World?

### 2.1. Global Drivers towards Circular Economy

What is the problem? The planet Earth is under severe stress due to imbalances in production, consumption, abuse and misuse of natural and man-made resources and poor climate control. Global food production will have to increase by at least 60% by 2050 [6]. The industrialized nations are resource intensive societies; thus, sourcing of alternative feedstocks can be a solution to find natural sources of heterogeneous compounds [7].

How should we approach the problem? The identified needs led to United Nations developing 17 sustainable development goals (SDGs) to protect the planet, end poverty, and ensure prosperity for all humanity. However, only 12 were directed to ensure the improvement of social and industrial behaviour towards a more responsible consumption, improvement of basic services, with a greener point of view, and better quality of life. Therefore, consumption reduction will lead to waste reduction, and several approaches should be done to stimulate the reuse and recycling of food products, improving the overall of food systems, from producer to consumer [8].

What do we want to achieve? Through the global drivers and the SDGs, humanity wants to achieve a global bio-based economy, and the European Union calculates that for each 1€ invested in EU-funded bio-economy research and innovation, approximately 10€ value added will be returned to bio-economy sectors by 2025 [6]. Furthermore, new knowledge and opportunities will be created as traditional chemistries will be superseded by new green chemistry practices and principles [9] and biorefineries will emerge for maximum resource re-utilization in the next few years. Therefore, food supply chain waste represents an interesting resource because of its high volume, chemical richness and heterogeneity [10].

### 2.2. Circular Economy

The European Environment Agency (EEA) described somewhere ‘stimulating resource-efficient, low-carbon economic and social development’ as an essential milestone to achieve by 2050, requiring a worldwide trend to explore possible paths for the transition from linear to circular economy (CE) business models. In a linear economy, the central economic development model is the raw materials that are taken, processed, consumed and disposed of as waste [11]. In the concept of a circular economy, recovery and valorisation of waste allows materials to be reused and put back into the supply chain, allowing economic growth from environmental losses [12].

So far, a lot and different CE studies have been published triggering the CE subject in a theoretical way, but also in practical way, with development and discussion of new approaches in different fields. Moreover, several countries have tried to apply a CE in closed environments, to integrate not only economic and environmental issues, but also the technological and social aspects, to develop implementation models and eliminate early problems that might occur in a future society scale up.

However, sustainable development requires balanced and simultaneous consideration of the economic, environmental, technological and social aspects of an investigated economy, sector, or individual industrial process, as well as of the interaction among all these aspects. Therefore, a CE is described by MacArthur et al. [13] as an efficient use of resources, which applies only green chemistry in the technological field, that will lead to the development of new business models and creation of innovative employment opportunities beyond other benefits.

But what are really the benefits of CE and how can these be assessed, as well as the potential negative effects? What should be transferred from theory to practice? What business models will help in this transition? These issues have been discussed in recent years all around the world. Several documents have already been published, which focus on recycling and recovery strategies all along the lifecycle of a product [14], but is this enough? A simple and incorrect approach to CE has been taken since only an approach to waste management has been considered, but such limited point of view leads to incorrect implementation procedures and therefore failure of this interdisciplinary method. The implementation of CE needs to be widely applied compared to how it is at this moment, since it involves recycling and reuse of resources, renewable energy production, but also the development of alternative solutions to the entire food supply chain. In addition, the interaction between the processes and environment in which the economy is embedded must be seen as an improvement of the total system, where the economic models must improve the economy and resource management [12]. Nevertheless, few studies measure effectively the “circularity” level of a product, a supply chain or a service, but only explore and analyse the application in different contexts [15]. Therefore, the CE involves a high complexity vision of sustainable development that should involve all kinds of thinkers and developers for a better application. Moreover, the involvement of industry, society and politics for improvement of services [13] will complete the energy cycle and maximize the ethical and economic value.

Therefore, the CE fits well in the future management of global resources, since it endorses the prevention use of virgin resources, optimizes the yields of reusable products through manufacturing improvement and generates lower amount of waste by promotion of loop closing[16].

#### Circular Economy Implementation Levels

Ghisellini et al. [12] carried out a large study on CE and was able to divide the subject into topics of interest. First, the CE was divided into two conceptual groups *CE models* and *CE principals*, and these were further divided in three study scales, implementation at micro level, meso level and macro level.

A circular economy at the **micro level** means that implementation occurs at a single company or consumer. The implementation in the production sectors, first requires adaptation procedures inside the company, and then entails cooperation between other companies throughout all supply chain. Since the reuse of secondary products (matter or energy), combined with implementing strategies improves process yields, decreasing the use of energy and water, creating new value from waste, leading to effective implementation of a circular pattern [17]. On the other hand, in the consumer sector, promotion of consumer responsibility is crucial for enhancing the purchase and use of more sustainable products and services; therefore, functional instruments for green consumption should be adopted. However, the micro level also covers the best use and recovery of resources; therefore, waste management has a high impact in CE, “*with emergence of new typologies of operators and processes, among which the so-called “scavengers” and “decomposers”, referring to companies capable to extract resources out of waste by applying innovative recovery technologies*” [12].

Scavengers and decomposers are fundamental pillars in the new drivers of CE. On the one hand, the first pillars are not only the collectors, but also the distributors of waste from other companies. These companies collect the remains of the primary raw materials and redistribute them again into the system. The second pillar are the decomposers, who turn the waste into secondary raw materials, developing new products for the supply chain and integrating new economic income via reused materials, through the application of recycling and green chemistry [14,18].

A circular economy at **meso level** refers to the development of eco-industrial parks where industrial symbiosis occurs. In this instance, industries will work together developing an exchange of resources, at different levels, such as, recirculation of water, energy and matter, achieving economical and environmental benefits. “*The essence of industrial symbiosis is taking full advantage of by-product utilization, while reducing residual products or treating them effectively*” [12,19].

The CE at **macro level** is a large overview, including the development of cities, provinces or regions, that will involve the integration and redesign of four systems: the industrial system, the infrastructure system, the cultural framework and the social system, connecting everything, leading to the formation of eco-cities through final application of several initiatives from the micro and meso levels.

What is important to remember about the CE? Is still in an early application stage. For now, it is a feasible application to replace the linear economy model. Therefore, the central pillars of CE are based on the recovery of waste, by increasing their time in the supply chain, increasing the economic value and releasing such products from primary processing, leading to the development of secondary processing for the delivery of new materials and/ or energy production. Novel and new processes and products should be developed to allow the utilization of virgin materials to be minimized and, in a second step, to maximize the use of the waste throughout the supply chain, by promoting circularity, thus closing the loop and decreasing the amount of noxious materials deposited in the environment [20].

## 3. Fruit Processing and By-Product Production

Food waste management is a critical issue for global food safety. Fruit processing industries are one of the main producers of food waste. In this instance, the Europe Commission prompted new targets on the reduction of fruit waste to stimulate Europe’s transition towards a CE [21]. Fruit processing industries produce a great number of consumer natural products such as juice, jams, salads or snacks, among others. Nevertheless, these activities generate large amounts of fruit by-products such as peel, seeds, pomace, bagasse, etc. However, these natural matrices still contain a high amount of bioactive compounds (BCs) with relevant chemical and nutritional value, mainly pectin, proteins, antioxidants and phenolic compounds, with beneficial effects on human health. Furthermore, these by-products are rich sources of complex polysaccharides, carbohydrates, fibre and vitamins. The recovery of these BCs is a challenging and important task for their return to industrial chains (commercialization); thus, they can be employed for the arising trends regarding human demand (Table 1).

Thus, a description of the main fruit by-products generated is required to understand the path that must be followed to reuse such products, as well as the development of new strategies towards a CE. 

### 3.1. Apple

According to FAOSTAT (2017) 366 million tons per year of apples are produced, with 3–4.2 million tons of apple pomace by-products produced worldwide. The apple pomace includes the peel, seeds, stem, cores and some edible parts (remnant), which traditionally are formed during apple processing. The high number of apple by-products induces different problems concerning transportation and disposal. However, these materials are good sources of polyphenols, mainly presenting in peel. The most identified BCs are hydroxycinnamic acids, catechin, quercetin and epicatechins. On the other hand, apple by-products contain high pectin levels which have gained relevant uses for different industrial purposes, such as biofuel precursors (bioethanol) and food ingredients (thickening agent, gelling agent and stabilizer) [22]. Additionally, some of these BCs have been applied as antioxidant extracts for beneficial uses in human health, mainly helping to restore intestinal functions and lower serum cholesterol levels [23].

### 3.2. Tomato

Tomato is considered a valuable crop and has its origins in South America. According to FAOSTAT (2017), the annual global production of fresh tomato is near to 242 million tons, making it one of the most important fruits cultivated worldwide.

Production of the industrial by-products of tomato is first generated during the transport and then during processing. The main by-products generated are seeds, peel and pomace, with a production of 4 million tons estimated per year [24], leading to major environmental problems due to their quick disintegration and production of bad odours though microbial promotion. However, tomato by-products are an important source of vitamins, minerals, proteins and other BCs, especially carotenoids such as β-carotene and lycopene. These phytochemicals confer a high nutritional value matrix, as well as beneficial bioactivities, mainly antioxidant activity. Additionally, β-carotene also has pro-vitamin A activity, which can protect the eyes from photooxidation, immune system functions and prevention of chronic diseases [25].

### 3.3. Banana

Banana is an important crop cultivated in tropical and subtropical weather, mainly Asia, South America and Africa. By-product production was estimated at 101 million tons in 2017, mainly peel, equivalent to 35–40% of the total weight of the fresh fruit [26]. Industrially, banana has been employed for the production of natural consumer products (chips, salads, jams or pure), while peel by-products are disposed as waste. Generally, the banana’s by-products (peel) are dispersed in the planting area as composting material. However, this activity has not been enough due to massive accumulation, promoting an adverse environmental risk. Furthermore, banana peels are characterized as a source of carbohydrates, vitamins and lignocellulosic compounds, as well as phytochemicals with antioxidant properties, pigments (flavonoids) and catecholamines. Currently, a large number of applications for these by-products have been reported, namely in bioethanol and biogas production and heavy metals removal [27].

### 3.4. Mango

Mango, also called the king of fruits due to its tasty flavour, attractive aroma, beautiful colour and nutritional value, is grown naturally in tropical regions, and its production reached about 45 million tons in 2017. Processing of mango leads to the production of significant amounts of by-products mostly peel and kernels (seed) representing around 24% and 40% of fresh weight, respectively. Mango kernels are characterized by a high carbohydrate and protein content and good profile of essential amino acids and lipids. In turn, the peel has shown to be a source of polyphenols such as flavonols, O-and xanthone C-glycosides, gallotannins and benzophenone derivates. These compounds have antioxidant, antifungal, antiproliferative, anti-atherogenic, anti-thrombotic and anti-inflammatory effects [28]. Additionally, mango peel has considerable levels of dietary fibre, which has been the target of some studies associated with its benefits, especially for the prevention of illnesses such as diabetes, gastrointestinal disorders, obesity and cardiovascular diseases [29], through production of functional flours with high levels of dietary fibre and antioxidant extracts.

### 3.5. Citrus fruits

The worldwide production of citrus fruits, including lime, lemons, mandarins and oranges, was estimated to be over 88 million tons in 2017 [22]. Citrus fruits are categorized as fruit with an acidic taste attributed to the high amount of citric acid, a powerful natural antioxidant. Normally, these fruits are consumed in the market as fresh fruit or as industrial products in juices, marmalades or flavouring. However, such industrialization produces a considerable quantity of citrus by-products, commonly peel. Only in the Europe Union, more than 10 million tons of citrus fruits are estimated to be produced, with a very low economic value. In recent years, citrus peels have been shown to retain a high concentration of polyphenols such as phenolic acids and flavonoids [30]. On the other hand, citrus by-products can be employed for the recovery of essential oils, principally D-limonene, a compound considered a natural antimicrobial with potential application in the food and pharmaceutical industries [31].

### 3.6. Pineapple

Pineapple is a tropical fruit with attractive sensorial and nutritional (vitamins, antioxidants and fibres) characteristics, complemented by a natural catalyser, called bromelain [32]. FOASTAT (2017) database presents an estimated production of 29.5 million tons per year, which are designated to industrial manufacturing of salads, essences, juices, jams and dehydrated fruit, amongst others, causing continuous by-product production. The generated pineapple by-products, including the crown, peel, bottom, stem and trimmings, are a huge problem because they represents almost 60% of the total fresh weight [33]. Pineapple stems and peel are highlighted for their bromelain content, which has been extracted for applications in several industries. In the past few years, the bromelain market for industrial applications has been growing due to biotechnological uses and due to their useful characteristics against chemical catalysers, making a safe and less costly option for industries. However, more recently, several works have described the high content of phenolic compounds and vitamin C in pineapple by-products and the possible production of antioxidant extracts from pineapple juices or the possible production of pineapple functional flours from pineapple stems and cores, due to the high content of dietary fibre that is complexed with BCs responsible for high antioxidant activity [34].

### 3.7. Grape

Grape is an important cash crop having a positive economic impact in the world, since almost 67% of the total grapes are destined for the wine and other alcoholic beverages [35]. As a result of this activity, according to Matharu et al. [10], around 5–9 million ton/year of grape pomace were produced in 2017 worldwide. This by-product is constituted mainly by skin and seeds, both well documented to contain a rich content of BCs such as phenolic acids, flavonoids, anthocyanins and proanthocyanidins. Some reports indicated that these BCs are of great interest for their pharmacological and biological properties, including cardioprotective, antimicrobial and antioxidant activity [36].

On the other hand, several wineries are applying the by-products generated during wine production in the production of flours and new pastas (substitution of wheat flour for grape flour), with biological activities associated [37].

### 3.8. Melon

Melon fruit is a juicy, tasty and delicious fruit well-accepted for its nutritive and medicinal properties and naturally low in fat and sodium, and it can be a vital source of minerals, dietary fibre, polyphenols and carotenoids [38]. Worldwide melon production is estimated at 49 million tons/year (FOASTAT 2017), and once more, industrial processing generates great quantities of peel and seed by-products with no value or any featured applications. Melon peel and seeds represent about 25% and 7%, respectively of the fresh total weight, meaning that more than 30% are by-products, generating great economical losses [39]. In addition, these by-products stand out because of the significant concentrations of BCs and have generated scientific interest due to their potential [40]. Furthermore, melon fruit contains a serine protease called cucumisin, which can be employed in food industries [41]. Thus, due to all this evidence, studies on melon by-products have been increasing, leading to the augmentation of economic value, but also enhancement of global issues.

## 4. Fruit By-Product Management: Valorisation of the Development of New Value-Added Products

Fruit by-products have been recognized for their relevant nutritional composition and biological activities with potential beneficial effects on human health. Nowadays, the CE has recognized the valorisation of fruit by-products as important for the recovery of BCs from food by-products, as well as the management of environmental impact prevention [43]. Fine chemistry coupled with the CE approach can maintain the same value of consumer products, as well as add value to by-products, retaining them within the industrial chain, aiming at the reduction of the generated waste. Thus, the application of new and novel methods achieves global competitiveness and sustainable growth by creating new business and jobs [44].

Nowadays, these matrices have been valorised through a fine chemistry approach to obtain new and diverse bioproducts based on use of non-toxic organic solvents and low energy consumption, which could help in the resolution of the global issues [45]. In the last ten years, there has been a number of broad research studies about recovery/production of pectin, enzymes, antioxidants and phenolic compounds through eco-friendly methods (Table 2), which shows the importance of the recycling and re-valorising of fruit by-products as great resources for future developments in the prevention of current problems. However, these BCs are present in high concentrations, may not be directly available and may require pre-processing steps for easier extraction.

### 4.1. Added-Value Products and Bioproducts Derived from Fruit By-Products

Depending on the fruit processing technology (e.g., drying and dehydration, pressing, fruit jams, canning, jellies), solid (e.g., pomace, pulp, peels, cores, seeds, and stems) as well as liquid (e.g., juices, wash water, chilling water and cleaning chemicals) by-product streams are produced [46]. Most of the processed fruits are highly fermentable and perishable, mainly because of high moisture (80–90%), free soluble sugars (6–64%) and crude protein (10–24%) contents. However, these by-products also retain a significant amount of BCs such as phenolic compounds, pigments, dietary fibre, essential oils, enzymes and organic acids, which can be extracted and exploited mainly as commercially valuable materials for food, pharmaceutical and biofuel industries.

### 4.2. Antioxidant Extracts

Food additives are molecules which are introduced in foodstuffs to carry out specific technologic functions and are divided into 6 groups: preservatives, nutritional additives, colouring agents, flavouring agents, texturizing agents, and miscellaneous agents, with specific expected outcomes in the food. The preservatives group are divided into three smaller functional groups, namely antimicrobials, antioxidants and anti-browning agents [59]. The antioxidant subgroup of additives is used to extend the shelf life of foodstuffs from the oxidative process that could result in degradation of food such as lipidic peroxidation, changed flavour, colour, nutritional value, texture, as well as creation of toxic compounds. For itself, antioxidant compounds are one of the most important conservation technologies used by the food industry with their main function being the prevention of oxidative processes [60].

Synthetic antioxidants such as butylated hydroxyanisole (BHA) and butylated hydroxytoluene (BHT) have been used widely as antioxidants in the food industry. Due to concerns over the safety of synthetic antioxidants [50,51,52] and to a shift in consumer interest in natural antioxidants (i.e., with less additives) [61] these synthetic antioxidants have been substituted by phenolic compounds, and subsequently, much of the research on natural antioxidants also focused on phenolic compounds. The structure of phenolic compounds is the key of their antioxidant activity (to scavenge free radicals, donate hydrogen atoms or electron, or chelate metal cations) [62]. The higher antioxidant activity of hydroxycinnamic acids compared to the corresponding hydroxybenzoic acids could be due to the CH=CH–COOH group, which ensures greater H-donating ability and radical stabilization than the –COOH group of hydroxybenzoic acids [63]. The case of flavonoids is more complicated than that of phenolic acids due to the relative complexity of these compounds [62].

Various recent studies demonstrate an improvement in total phenolic content and antioxidant activity after incorporating antioxidant by-product extracts. For instance, the addition of 500 mg of pomegranate peel extract in 100 mL of apple juice decreased the relative free radical concentration by 5%, while the total phenolic content increased by 19%, when compared to the control [64]. Adiamo et al. [65] showed that the addition of orange peel and pulp phenolic extracts in carrot juice enhanced its phenolic content and antioxidant activity; however, higher amounts of phenolic and antioxidant activity were obtained in juice containing peel phenolic extract. Kulichová et al. [66] evaluated the impact of phenolic extracts from oil cake of white grapes in apple and grape juices, concluding that the juice with 1 g/L of extract increased in antioxidant capacity and total polyphenol content two-fold [66]. Larrosa et al. [67] reported that inserting an phenolic-enriched extract from blanched artichoke by-product in a tomato juice resulted in higher antioxidant activity (measured by in vitro methods) and, consequently, a longer shelf life for the tomato juice. Furthermore, Roldan et al. [68] showed that onion by-products (namely onion peel and stem) are a potential antioxidant additive due to their antioxidant and anti-browning properties.

Regarding grape by-products, some studies have proposed the direct use of grape pomace as an additive in wine production, and Pedroza et al. [69] demonstrates the potential of dehydrated grape skin powder as a raw additive for releasing a valuable amount of pigments and aromas in wines. In turn, natural pigments due to the high concentration of anthocyanins increase the natural antioxidant and antimicrobial contents towards food additive application [70].

Antioxidant extracts are frequently applied in other foodstuffs, in meat [71,72,73], in fish [61,62,63], in dairy [61,71,72,73] and in bakery products. Antioxidant extracts proved to be efficient in delaying oxidation processes and microbial spoilage in animal and dairy products due to the antioxidant activity of phenolic compounds. The application of antioxidant extracts in bakery products such as bread, cookies and cakes have been widely reported [74]. Pathak et al. [75] showed that the incorporation of 3 g of mango peel powder in 100 g of whole wheat bread increased the total phenolic content from 220 to 507 mg of gallic acid equivalence per 100 g, as well as increasing antioxidant activity (using FRAP and DPPH method). Maner et al. [76] replaced a portion of white wheat flour with grape pomace powder (5 g/100 g) in cookies and obtained the best organoleptic scores and increased total phenolic, flavonoid, tannin and anthocyanin contents 2.3-, 2.0-, 1.3- and 12.5-fold, respectively, compared to control cookies.

#### 4.2.1. Phenolic Compounds

In a general way, the fruit by-products have high accumulation of phenolic compounds than the edible part, which means a loss of an expressive biological value [49]. Structurally, phenolic compounds are constituted by an aromatic ring with one or more hydroxyl substituents. Phenolic compounds can range from simple phenolic molecules to highly polymerized compounds, and regardless of the structural diversity, the group of compounds are often referred to as “polyphenols”. The classifications of polyphenols are based on their chemical structure as phenolic acids, tannins, flavonoids, lignans and stilbenes, among others, naturally occurring in free or bound form [77]. Phenolic compounds have multiple biological effects, including antioxidant, anti-inflammatory, antimicrobial activity, and they may prevent degenerative, cardiovascular and neurodegenerative diseases, as well as some types of cancers. Between fruit by-products, the content and types of polyphenols are different, and nowadays, in the CE context, polyphenols have been recovered from these organic materials mainly using green extraction methodologies to avoid environmental pollution and economic expenses as well as to minimize the toxicity of the final products. Apples, grapes and citrus fruit by-products are a well-known source of polyphenols that have been extensibility studied [31]. Additionally, some research studies have shown that peels, in the majority of fruits, have a higher content of polyphenols when compared with pulp, which is expected since the polyphenols are the main compounds responsible for the protective role against insects and microorganisms [78].

Therefore, hydroxycinnamic acids (chlorogenic and caffeic acid), flavonols (quercetin), flavanols (catechin, epicatechin and procyanidins), dihydrochalcone (phloridzin) and anthocyanins (cyanidin glycosides) are the major constitutes of apple pomace, with polyphenols being the major BC responsible for the antioxidant activity of this by-product [79]. Furthermore, grape pomace is rich in proanthocyanins, phenolic acids (hydroxybenzoic and hydroxycinnamic acids) and flavonoids (flavanols, flavonols and anthocyanins) [80]. Citrus by-products have a high content of phenolic compounds, such as naringin, hesperidin, narirutin and neohesperidin. Particularly, citrus peels are an abundant source of natural flavonoids and polyphenols, with some studies reporting the content of some lemon, orange, and grapefruit peel to be about 15% higher compared to the edible portions [81].

#### 4.2.2. Pigments

Carotenoids are another class of phytochemicals present in plants and constitute common pigments, such as β-carotene (orange), lutein (yellow) and lycopene (red). These phytochemicals occur in several fruits, and subsequently, after industrialised fruit processing, these compounds are still preserved in the by-products. The compounds are generally tetraterpenoid pigments (C40) and can be split into two classes based on the presence or absence of functional groups. For instance, β-carotene and lycopene do not have any functional group, but the molecules from the xanthophylls, lutein and zeaxanthin contain oxygen as their functional group.

The most known biological activities of carotenoids are antioxidant and pro-vitamin A activity [82]. Tomato by-products are a rich and cheap source of carotenoids, such as lycopene and β-carotene. In humans, the most well-known function of carotenoids is an enzymatic conversion in the body to vitamin A.

Vitamin A is an essential molecule involved in several physiological processes such as growth, reproduction and immune function enhancement. Moreover, carotenoids may also play an important role of photoprotection in the eye and prevention of chronic disease [83]. On the other hand, citrus by-products are one of the most complex sources of carotenoids, with a large diversity among the different species and cultivars in terms of type. It is an interesting fact that citrus fruit contain a group of specific C30 apocarotenoids, β-citraurin, which provides the intense orange-reddish colour to the peels. Interest in carotenoids is increasing due to the search for natural colourants, mainly by the food industry, since the European Union is restricting the application of synthetic colourants. Scientific research has been carried out on the extraction of such compounds, not only as natural colourants, but also as functional ingredients, having a multi-purpose in foods industrial chains. However, despite the correct application of carotenoids, these compounds are very unstable, as well as insoluble in water, leading to low bioavailability. The instability and insolubility in water can be addressed by the formulation of water-dispersible market products, such as colloidal suspensions, emulsions, or dispersions in suitable colloids. Moreover, in recent years, a great biotechnological effort has been centred on carotenoid encapsulation and nanoencapsulation to incorporate these beneficial compounds into food matrices. On the other hand, anthocyanins are the largest group of secondary plant metabolites, water-soluble pigments widespread in the plant kingdom, touching practically all visible spectra, from orange and red colour through to purple and blue colour [84]. Chemically, anthocyanins are flavonoids that naturally occur as glycosides of flavylium or 2-phenylbenzopyrylium salts and are mostly based on six anthocyanidins: cyanidin (50%), pelargonidin (21%), peonidin (12%), delphinidin (12%), petunidin (7%) and malvidin (7%) [85]. The sugar bounds, commonly glucose, rhamnose, galactose or arabinose, can be in mono- or disaccharide units, and they may be acylated with a phenolic or aliphatic acid [86]. Among the best sources of anthocyanins are grapes and several red fruit berries. Generally, one of the main approaches used in food industries is the production of natural colourants through extraction and isolation from whole fruit tissues.

#### 4.2.3. Dietary Fibre

In recent years, consumers have become more interested in a diet rich in fruits for a healthy lifestyle, not only because of the low calories, sodium and fats but also because of the richness in minerals, BCs and dietary fibre (DF). DF intake has shown several health benefits, including the regulation of intestinal transit, risk reduction of obesity, diabetes and cardiovascular diseases, as well risk reduction of hyperlipidemia, hypercholesterolemia and hyperglycemia, because it modulates food ingestion and influences the digestion, absorption and metabolism of nutrients [87]. Fruit by-products have been discovered as a novel source of DF and are the main source of pectin, gums and mucilage, which can be incorporated into market food products. Normally, DF from cereals is used more frequently than those from fruits; however, fruit fibres have more quality due to their higher total and soluble fibre content, water and oil holding capacity and colonic fermentability, as well as their lower phytic acid content and caloric values. Based on chemical, physical and functional properties, DF can be classified mainly in two groups: water-soluble and water insoluble. Water-soluble pectin, gums, inulin-type fructans and some hemicelluloses, which dissolve in water by forming a viscous gel and are resistant to digestion in the small intestine but easily fermented by the microbiota of the large intestine. The fermentation of water-insoluble DF is very limited in the human gastrointestinal tract, due to the insoluble carbohydrates structures, however, severely limited in the human gastrointestinal tract [88].

Pectin is a major component of all plant matter and mainly consists of D-galacturonic acid and monosaccharides including L-rhamnose, L-arabinose, D-galactose and D-xylose [89]. Commercially, pectin is extracted from different plant sources, the most common are from apple and citrus pomace. Citrus fruit by-products are considered an excellent source of dietary fibre, including both water-soluble (pectin and gums) and water-insoluble (cellulose and hemicelluloses) groups. Pectin is the predominant type of water-soluble fibre in citrus (65–70% of the total fibre) and is considered as one of the most reasonable ways for citrus pomace or peel valorisation. Apple pomace consists of 10–15% of pectin on a dry weight basis with superior gelling properties, compared to citrus pectin, but with a brown hue that may limit its incorporation into light-colour foods. To address these problems, scientists have been redirecting their interest to by-product valorisation from fruit processing industries, searching for new, different sources of pectin, such as peach pomace, banana, papaya, pomegranate and melon peel. There is an increasing interest in pectin because of its potential to lower blood cholesterol levels and triglycerides; pectin also affects glucose metabolism by lowering the glucose response curve. The main use of pectin is as a food additive because of its specific gelling properties and fat replacement. Some researchers have been studying the implementation of watermelon and melon dry peels in the production of flours to produce cakes, to improve the functional and nutritional characteristics through supplementation, as well as a providing a cheap surrogate for commercial corn flour [90].

#### 4.2.4. Essential Oils

Essential oils (EOs) are volatile, natural, complex compounds characterized by a strong odour and are formed by plants as secondary metabolites. In nature, EOs play an important role in the protection of the plants as antimicrobials, antifungals and insecticides, as well as providing protection against herbivores by reducing their appetite for such plants. The EOs may also attract some insects to promote pollen and seeds dispersion, or be used otherwise, to repel undesirable bugs [91]. The main BCs that have been found in the EOs are terpenes, phenylpropanoids, aldehydes, alcohols and ketones, and several studies have related them with their antioxidant, antimicrobial and antifungal activity [92]. Due to their antimicrobial properties and pharmaceutical and food use, studies in the recovery of EOs from fruit by-products have been more widespread in industrial sectors [93].

EO has been used in several meat products as a preservative because these molecules could provide antimicrobial properties against several food spoilage bacteria. Furthermore, EOs are a prominent alternative to synthetic chemical antioxidants and antimicrobial molecules because synthetics have been limited due to their undesirable aspects including their carcinogenicity effect and acute toxicity [94]. Fruit by-products, mainly seeds and skins, still preserve a significant concentration of EO, representing novel sources for their recovery. Citrus peels are a potential source of EO, and it is widely used in alcoholic beverages, confectioneries, soft drinks, perfumes, soaps, cosmetics and household products owing to its attractive aromatic flavour. On the other hand, they can be applied to extend the shelf life of foods, due to their broad spectrum of antibacterial activity. Citrus EO is characterized by its key component, D-limonene, a commercial terpene mostly obtained from orange peel [95]. In recent years, a valorisation of fruit by-products for recovery of EOs has been performed to minimize by-product accumulation, as well as obtain value-added by-products. EOs have been the target molecules due to their potential applications in food and pharmacological uses, supported by their bioactive properties.

### 4.3. Enzymes

Nowadays, enzymes are target biomolecules due to their favourable industrial applications [96]. These biomolecules are catalysers and can initiate an appropriate reaction without extreme conditions of temperature, pH and pressure, which results in contamination reduction and the saving of money for industries. Currently, under a valorisation context, enzymes have been recovered from fruit by-products. Some research has been carried out regarding this subject, such as that of Campos et al. [33]. They employed pineapple by-products for bromelain extraction using green technology and obtaining high recovery and purification percentages.

Bromelain is a proteolytic enzyme which has high specific activity and is employed in the pharmaceutical, cosmetic, nutraceutical and food industries [97]. Moreover, some researchers have mentioned the extraction of cucumisin, a proteolytic enzyme from melon pulp, which has caseinolytic activity and can be used in the dairy and cheese making industries as a replacement for traditional animal rennet [98]. Additionally, this enzyme can also be extracted from melon peel, which has shown caseinolytic activity through skimmed milk-clotting activity. This evidence provides sustainable information to maintain research to valorise fruit by-products by extracting value-added molecules. On the other hand, enzymes can also be produced using biotechnological processes.

Solid-state fermentation is a bioprocess well-documented in the production of microbial enzymes such as cellulase, xylanase, lignin peroxidases, tannase and lipase among others [68,69]. Presently, there is a lot of information regarding food by-product employment for enzyme production/extraction, however, there are few reports for the implementation of specific fruit by-products for enzyme production. In turn, pomegranate peel powder was employed for ellagitannase and cellulase production by *Aspergillus niger* through solid-state fermentation [99].

The lack of applicable sustainable studies in this field and the increasing availability of fruit by-products leave ample room for exploration over the next few years in the field of the production of commercial value-added biomolecules. The new studies, more than bringing novelty, should contemplate global environmental issues to wisely achieve the Europe Commission objectives and Sustainable Development Goals (SDG).

## 5. Economic and Business Approach

Changes are taking place worldwide in business strategy as industries face increasing pressures from economic crises, resource scarcity and pollution. Many different approaches to sustainability have been explored for the manufacturing industry, but despite efforts to reduce their carbon footprint, a large amount of enterprises continue to operate based on a take-make-dispose rationale [100]. The characteristic of a product directly influences the way the entire value chain will be constructed and managed. Therefore, processing design has a crucial role in supporting closed-loop supply chains and shared ownership models for sustainability.

Several pragmatic difficulties require specialists from various areas, including sciences, building, financial matters and administration, in order to address and resolve them. The CE must give financial motivations to guarantee that post-used items are reintegrated upstream into the assembling procedure. One of the obstacles that the circular economy faces is the cost to produce and implement reliable and comparable processes and technologies [13,100].

The “biorefinery” concept is still in early stages and it encompasses a sustainable processing of biomass into a wide range of marketable products, including fuels. Initially, the complex polymers that constitute biomass can be broken down into their component building blocks (carbohydrates, proteins, fatty acids) and subsequently converted into value-added products (e.g., enzymes and nutraceuticals) [24]. Therefore, a large spectrum of commercially important products such as biofuels, enzymes, organic acids, biopolymers, nutraceuticals and dietary fibres have been developed from the bioconversion of food industry waste.

Portugal is on the Atlantic coast of Europe and the Portuguese population generally follows a Mediterranean diet, fruits, vegetables, seeds, fresh fish and shellfish, as the main foods in the daily meals. The lack of time and busy life of the consumers led the Portuguese industries to adapt and develop more practical and easy ways to consume this kind of food. Therefore, in the past few years, several companies have arisen and the ones with greater impact on this field are those that process fruits for daily consumption; fresh, cut, ready-to-eat fruit has increased, as well as dehydrated fruit snacks. Yet, in the European market, the diversification of fruits consumed has increased, mainly tropical fruits such as natural mango, papaya and pineapple, in addition to banana.

Trade Map is a website of trade statistics for international business that shows grouped data about exportation and importation worldwide, as well as the traded economic values. This database shows data regarding the importation of tropical fruits, and in Europe, the biggest importer was the Netherlands with 1.02M tons. Another example, Portugal, registered an importation value in 2017 of 103,122 tons of tropical fruits (figs, pineapples, avocados, guavas, mangos and mangosteens) in fresh and dried state, representing 0.9% of the total world’s importation, making it the 20th largest importer on the main list, with a value of 107 M€ and leading to emission of 50.3 thousand tons of CO_2_. The main exporters to Portugal were Spain, Costa Rica and Brazil. As described before, a share of the imported fruits is used for processing, which means that part of the economic value is lost.

An assessment of agricultural residues and the agro-bioenergy co-products potentiality shows the suitability of production of second-generation biofuels or/ and high value-added co-products through an integrated biorefinery approach. Therefore, the main challenge for big players and stakeholders is to transform complex, heterogeneous and disposable biomasses into valuable and marketable high value-added products. However, an efficient conversion process depends transition from traditional process into innovative, green chemistry chain, as well as the development of adapted technological systems, in which process conversion, energy recovery and bio-based products are all integrated within an eco-friendly and efficient biorefinery system [101].

As introduced before, in the Portuguese industrial environment there are a very important group of fruit processing industries that are dealing with tons of fruit waste every day and this situation becomes more problematic when it involves imported fruits, since they already have an additional cost associated, as well as the emission of CO_2_. The implementation of downstream processes within industrial facilities will allow to these companies the development of new products, incorporating high economic value from the initial raw materials, being more sustainable.

### 5.1. Case Study: Pineapple Industrialization Approach to the Circular Economy

Pineapple industrialization occurs for different kind of industries, mainly food processing industries, but also enzyme industries. The authors of this chapter identified three groups where the main difference is the kind of processing applied to pineapple. The first group (G1) is a group that includes the fruit that endures small processing treatments such as crown detachment and peel and core removal. This processing occurs to produce ready-to-eat fruit and, in a later stage, could undergo a second treatment for producing dehydrated, crystalized and/or canned fruit. In the second group (G2) the fruit is subject only to the detachment of the crown, and the peel is removed, but at a later stage, undergoes hard processing (trituration, pressing, etc.) for the production of puree or pomace, pulps, jams, fruit concentrate, juice concentrate, among other products. The third group (G3) is the enzyme industry that detaches the pineapple crown followed by extraction of the natural bromelain from the pineapple fruit and stem.

**Figure 1:**
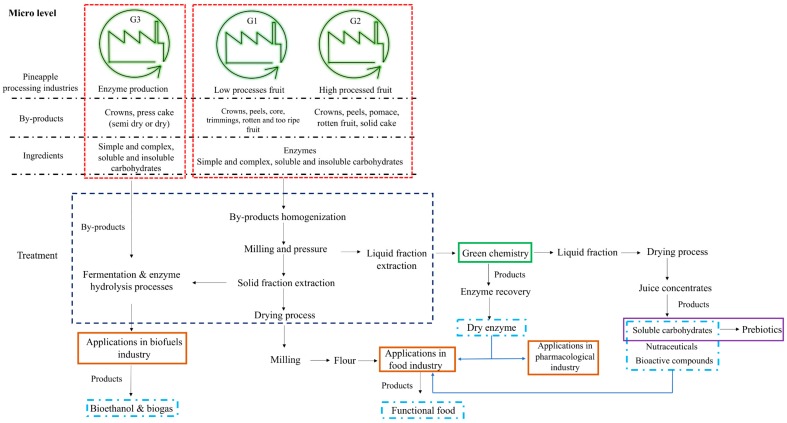
Implementation of circular economy at a micro level for pineapple processing industrialization.

Figure 1 describes the type and amount of by-products generated in the three groups of pineapple industrialization processes, and it is possible to observe that what is waste for one industry could be the main product for another industry. However, until now it has not been possible to join these industries because of the hard chemistry applied to some processing, which cannot be present in other final products.

The application of green chemistry to the extraction of natural enzymes can lead to the release of secondary raw materials for other industries, leading to the development of other foods and decreasing the number of by-products generated. Therefore, in this chapter, the authors will give a practical example of a micro and meso level implementation of the circular economy, by using real numbers and amounts of raw materials and the economic value of each process of the Portuguese reality.

#### 5.1.1. Micro Level Implementation

Implementation at a micro level requires implementation of each industrial facility; therefore, an adapted downstream process will be applied. Figure 1 describes each group that processes pineapple daily, and each type of waste generated. It is possible to see the kind of valorisation applied to each by-product. Therefore, from the G1 and G2, it is possible to obtain a mix of by-products that can be mixed to increase the total biomass generated. Considering a sustainable approach and low-cost processing, all fresh biomass should be mixed and homogenized. Liquid extraction should also be applied for separation of liquid and solid biomass. The solid fraction (or press cake) of pineapple has a high content of insoluble lignin but also cellulose and hemicellulose. This fraction could be reintroduced in the process to undergo solid state fermentation and enzymatic hydrolyses for biofuel production. The residual biomass would have similar uses to that previously attributed to the press cake, animal feed. The energy produced can be restored or sold or be applied to the energy maintenance of the industrial facilities, but, on the other hand, a functional ingredient can be developed from the press cake, which can be dried and powdered, for pineapple flour production. The liquid fraction of pineapple is full of BCs, but the most important molecule with the highest market value is bromelain; therefore, a green and low-cost separation process should be applied to separate this fraction [33].

The pressed liquid fraction should be transferred to a container, and usually, microfiltration or ultrafiltration can be applied to reduce the amount of water and sugars in the enzymatic fraction [102]. Hence, two liquid fractions are formed, having different applications: first, the enzymatic fraction (dried through freeze-drying), and second, the remaining liquid. The remaining liquid has a high concentration of simple and complex sugars, polyphenols and vitamin C, and can be dried through spray-drying and used as a juice concentrate (low market value) or can be applied as a functional ingredient by a promotion of antioxidant capacity or prebiotic activity (higher market value).

The G3 of pineapple processing generates two types of waste, which can be secondary raw materials, the press cake that can be directed to biofuels or flour production, as explained before. Generally, the liquid fraction of these industries cannot be applied to the food industry because of the content of hard chemistry used in the previous separation process.

As is noticeable in the above description, several advantages are associated with the micro approach for implementation of the circular economy, mainly through the development of new products that can be delivered to the market or applied in each industry. The main advantage associated is the increase of the economic revenue coming from initial raw materials—pineapple. But, there are some disadvantages, such as, the initial investment associated for the equipment implementation, the increase of human resources, the continuous maintenance of the process and the development of a new products with the different market channels.

#### 5.1.2. Meso Level Implementation

**Figure 2:**
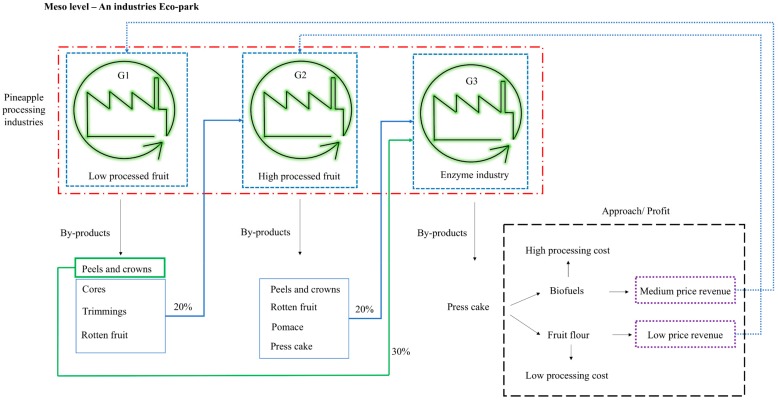
Implementation of circular economy at a meso level for pineapple processing industrialization.

The meso implementation requires the development of an industrial eco-park where the different groups of pineapple processing are in the same zone; therefore, the by-products generated by each industry will be transferred between industries, leading to the circularity of pineapple by-products. In Figure 2, it is possible to see that the by-products from G1 of pineapple processing can be used in part by G2, and the remaining by-products from G1 and the by-products from G2 can all be applied to G3 (enzyme extraction).

All industries have something in common, the need of raw material—pineapple. The different group of industries have different daily requirements of pineapple, but if each group needed entire pineapples (100%) as raw material, in total they would therefore require three times the weight of pineapple (300%). When a meso implementation is applied, G1 will use the entire (100%) pineapple but will generate 50% waste. G2 also need entire (100%) pineapples, but in this case, they can use processed pineapples cores, trimmings and overripe pineapple originating from the processing of G1; hence, at this point, G2 may receive 20% of their pineapple as by-products from G1 and just need to buy a further 80% pineapple fruit. G3’s industrial process usually uses the entire (100%) pineapple, but if they receive 30% peel (total from G1 and G2) and 20% pomace, overripe fruit and rotten fruit, this gives a total of 50% of secondary raw material.

Considering these values, G3 receives 50% of their pineapple by-products from the other industries, therefore, G3 only needs to buy 50% of their pineapple to fulfil the required need for their industrial production. However, one major by-product produced by the three industries groups—press cake. This could be used in a common industrial facility, for biofuel production providing energy to all industries.

As advantages, there is a reduction of the total need of raw material from 300% to 230% with the meso approach, meaning a saving of 70% of pineapples that could be directed to consumption. Furthermore, this approach leads to a reduction in the total by-products generated, since with current production, a total of 164.2% by-products are generated (106% peels, cores and crowns and 58.2% pomace), and with this implementation, two hypotheses can be suggested. From the liquid fraction, no waste is generated, everything is valorised; from the solid fraction (press cake), it is possible to produce biofuels, leading to the generation of energy and 20% (dry mass) of biomass (waste). On the other hand, the press cake can be totally dried, powdered and be used to produce a new product—pineapple flour. Pineapple flour is full of dietary fibre and complex soluble sugars. This second waste approach is not yet produced but has low-cost production, closing the loop of pineapple industrialization and achieving zero waste.

In the current reality of developing countries and considering the circular economy approach and implementation, as well as the economic and business approach, the more feasible approach for implementation is micro implementation because of the on-site application of CE.

## 6. Conclusions and Future Perspectives

Waste management is an issue of increasing importance for the food industry, which needs an overview of the whole food system to take immediate actions towards improvement. The CE has arrived to tackle this significant issue and to finally transform the food system into a more sustainable and environmentally friendly system, through the ‘*stimulation of resource-efficiency, low-carbon economy and social development*’ as an essential milestone to achieve by 2050.

The food processing industries produce large amounts of food waste, with very low reuse of the regenerated by-products, and this is more noticeable for fruit processing industries. Abundant agro-industrial by-products have been studied as a low-cost material for the generation of value-added products and energy production as a part of a biorefinery product portfolio (main goal—production of biofuels).

Integration of the valorisation concept allows the conversion of fruit waste into high-value products with relevant potential applications for human consumption, such as extraction of specific molecules and the production of antioxidant extracts and functional flours. Such transformation requires food, nutraceutical and pharmaceutical industries to open the doors to the improvement of the biological activities of current products, as well as the development of novel products.

The pineapple case study developed in this manuscript is the clear evolution of society towards a more sustainable future, where all foods, by-products and waste are reused until the very end. This valorisation approach not only addresses environmental issues but also creates an opportunity to build a multi-million-dollar industry for the manufacturing of new products, giving a glimpse of the future in the developing of closed-loop processing systems, encouraging CE and generating wealth from waste.

## Figures and Tables

**Table 1:** Summary of current potential applications of fruit by-products towards industrial purposes.

Source	By-Product	Value-Added Product	Industry	Reference
Sweet lime	Peel	Bioethanol and enzymes	Biofuel	[22]
Melon	Peel	Pectin	Cosmetic and pharmaceutical	[23]
Tomato	Peel	Lycopene	Pharmaceutical	[25]
Banana	Peel	Bioethanol	Biofuel	[26]
Pear	Sheets	Fiber, minerals and vitamins	Food suppliers	[29]
Pineapple	Stem, barks and leaves	Enzyme (bromelain)	Meat and pharmaceutical	[32]
Pineapple	Peel and core	Enzyme (bromelain)	Meat and pharmaceutical	[33]
Grape	Pomace skin	Fiber and antioxidant	Food suppliers	[35]
Peach and plum	Bagasse	Antioxidants	Nutraceutical	[36]
Melon	Peel and seeds	Antioxidants	Nutraceutical	[39]
Melon	seeds	Essential oils	Food suppliers	[40]
Melon	Pulp	Enzyme (cucumisin)	Dairy	[42]

**Table 2:** Examples of bioproducts obtained from valorization of some fruit by-products.

Fruit	By-Product	Methodology	Bioproducts	Reference
Banana	Peel	SSF by *Aspergillus niger*	Citric acid	[27]
Mango	Peel	Autoclave	Pectin and polyphenols	[28]
Tomato	Bagasse	Ultrasonic bath and high-performance homogenizer	Polyphenolic Antioxidants	[47]
Orange	Peel	Steam explosion	Limonene	[48]
Grape	Skin	SSF	Polyphenolic antioxidants	[49]
Grape	Bagasse	SSF	Polyphenolic antioxidants	[50]
Orange	Peel	Enzymatic and chemical hydrolysis	Pectin and Limonene	[51]
Passion fruit	Peel flour	Aqueous two phases systems	Polygalacturonase	[52]
Melon	Seeds	Extraction with n-hexane	Antioxidant oils	[53]
Watermelon	Seeds	Ultrasound treatment	Fatty acids, tocopherols	[54]
Avocado	Peel	Ethanolic extraction	Phenolic antioxidants	[55]
Pomegranate	Husk	SSF	Ellagic acid	[56]
Papaya	Peel	Extraction with n-hexane	Carotenoids, anthocyanins	[57]
Olive	Pomace	Multi-frequency Multimode Modulated ultrasonic	Phenolic antioxidants	[58]

Notes: Abbreviations: Solid State fermentation–SSF.
